# Inhibitor of DNA binding 1 in lung cancer and the tumor microenvironment: a testable hypothesis for malignant pleural effusion formation

**DOI:** 10.3389/fimmu.2026.1743032

**Published:** 2026-07-22

**Authors:** Yan She, Yike Du, Zhijun Chen

**Affiliations:** 1 Department of Cardiothoracic Surgery, Zhoushan Hospital, Zhoushan, Zhejiang, China; 2 Department of Cardiothoracic Surgery, Jiamusi University, Jiamusi, Heilongjiang, China

**Keywords:** ID1, immune regulation, lung cancer, malignant pleural effusion, molecular mechanisms, tumor microenvironment

## Abstract

Inhibitor of differentiation 1 (ID1) has emerged as a crucial regulator in the pathogenesis and progression of lung cancer, influencing various aspects of tumor biology, including tumor cell proliferation, invasion, metastasis, and immune modulation, within the complex tumor microenvironment. This review comprehensively examines the molecular mechanisms by which ID1 contributes to lung cancer, emphasizing its role in driving these processes. Despite significant advancements in lung cancer biology, the interactions between tumor cells and their surrounding microenvironment remain incompletely understood. This presents challenges for developing effective therapeutic strategies. We then examine the pathophysiology of malignant pleural effusion (MPE), a devastating complication of advanced lung cancer. Based on the striking mechanistic overlap between ID1-driven processes and the core drivers of MPE—vascular hyperpermeability, immunosuppression, and tumor cell seeding—we propose a unifying, testable hypothesis that ID1 may act as aputative key orchestrator of MPE formation, a proposition that currently awaits direct experimental validation. While direct evidence is currently lacking, we outline indirect clues and propose specific experimental approaches to test this hypothesis. By integrating recent findings from both basic research and clinical studies, this article emphasizes ID1 as a pivotal mediator in lung tumorigenesis and microenvironment remodeling. It also highlights its potential as a novel biomarker and therapeutic target, aiming to promote the development of improved diagnostic and therapeutic approaches for lung cancer, particularly in addressing challenges related to tumor progression and MPE.

## 1 Introduction

The inhibitor of DNA binding 1 (ID1) protein, a member of the helix-loop-helix (HLH) transcription factor family, has emerged as a pivotal regulator in the pathogenesis and progression of various malignancies, including lung cancer (1). ID1 functions primarily as a transcriptional regulator by negatively modulating the activity of basic HLH transcription factors, thereby influencing gene expression patterns critical for cell proliferation, differentiation, and survival (2). In non-small cell lung cancer (NSCLC), the expression of ID1 is notably elevated, correlating with metastatic disease and advanced stage, suggesting a link to tumor progression (3). Mechanistically, ID1 modulates critical oncogenic pathways such as epithelial-mesenchymal transition (EMT), a fundamental process that enhances cell motility and invasion, thereby driving tumor metastasis. Evidence from diverse malignancies, as detailed in later sections, supports the conserved role of ID1 in promoting EMT and metastasis. ID1 is also implicated in promoting vasculogenic mimicry, a phenomenon where cancer cells form vessel-like structures independent of endothelial cells, facilitating tumor vascularization and progression (4). These multifaceted roles establish ID1 as a crucial molecular player in tumor pathogenesis.

Given this functional versatility, it is within the lung tumor microenvironment (TME) that ID1 exerts its most profound impact on cancer biology. The TME in lung cancer is a highly dynamic and heterogeneous milieu (5) composed of cancer cells, stromal cells including fibroblasts and endothelial cells, immune infiltrating cells, extracellular matrix components, and soluble factors. This complex ecosystem profoundly influences tumor progression, metastasis, and therapeutic responses (6, 7). ID1 exerts significant regulatory effects within the TME by modulating cell-cell signaling pathways and immune cell functions (8–12). ID1 is a well-documented key regulator of tumor angiogenesis and a potent modulator of immunosuppression within the TME. The interplay between ID1 and TME components thus represents a vital axis influencing lung cancer progression and therapeutic resistance.

Malignant pleural effusion (MPE) is a frequent and debilitating complication in advanced lung cancer, occurring in approximately 50% of lung cancer cases and signifying a poor prognosis with median survival often limited to months (13). MPE formation results from complex interactions between tumor cells, pleural mesothelial cells, immune components, and the pleural vasculature, leading to increased vascular permeability, angiogenesis, and fluid accumulation. Despite its clinical significance, the precise molecular mechanisms orchestrating MPE development remain incompletely understood. Emerging evidence indicates that factors such as vascular endothelial growth factor (VEGF), inflammatory cytokines, and extracellular matrix remodeling enzymes contribute to MPE pathophysiology (14–18). Clinical studies have also demonstrated that molecular alterations including driver mutations (e.g., ALK, ROS1, T790M) and PD-L1 expression correlate with MPE development, underscoring the involvement of oncogenic signaling and immune modulation in effusion pathogenesis (13). Furthermore, tumor marker testing in the diagnosis of MPE significantly enhances diagnostic accuracy and prognostic assessment capabilities through the high specificity of markers like CEA and the utilization of multi-marker combination strategies (19). Therefore, understanding the molecular drivers of MPE is critical for advancing diagnostic and therapeutic strategies.

Within this context, the multifaceted regulator ID1 emerges as a molecule of particular interest. It is noteworthy that the established roles of ID1 within the lung TME exhibit a striking mechanistic overlap with the core pathological processes driving MPE. ID1 is a well-documented driver of aggressive traits in lung cancer: it promotes tumor cell proliferation, invasion, and metastasis through mechanisms such as facilitating epidermal growth factor receptor (EGFR) signaling (3), inducing EMT to enable liver colonization (20), driving angiogenesis in correlation with VEGF levels (21), promoting lymph node metastasis via BMP signaling (22), and shaping an immunosuppressive TME, as exemplified by its role in KRAS-mutant LUAD where its downregulation sensitizes tumors to immune checkpoint blockade (23). Meanwhile, MPE pathogenesis is fundamentally driven by three interrelated processes: vascular hyperpermeability (largely VEGF-mediated), an immunosuppressive microenvironment, and pleural seeding by tumor cells. This striking parallel raises a critical question that lies at the heart of this review: Could ID1, through these well-established functions in primary lung tumor biology, contribute to the pathogenesis of MPE? Addressing this question in Section 5, we synthesize the available evidence and present a testable framework that links ID1-driven processes to the pathophysiological pillars of MPE—vascular hyperpermeability, immunosuppression, and pleural tumor seeding—while critically assessing the evidence gaps and proposing experimental strategies for future investigation. It is crucial to emphasize that, while this hypothesis is mechanistically plausible, direct experimental or clinical evidence definitively linking ID1 to MPE formation is currently lacking, making this a testable hypothesis rather than an established mechanism.

**Figure 1:**
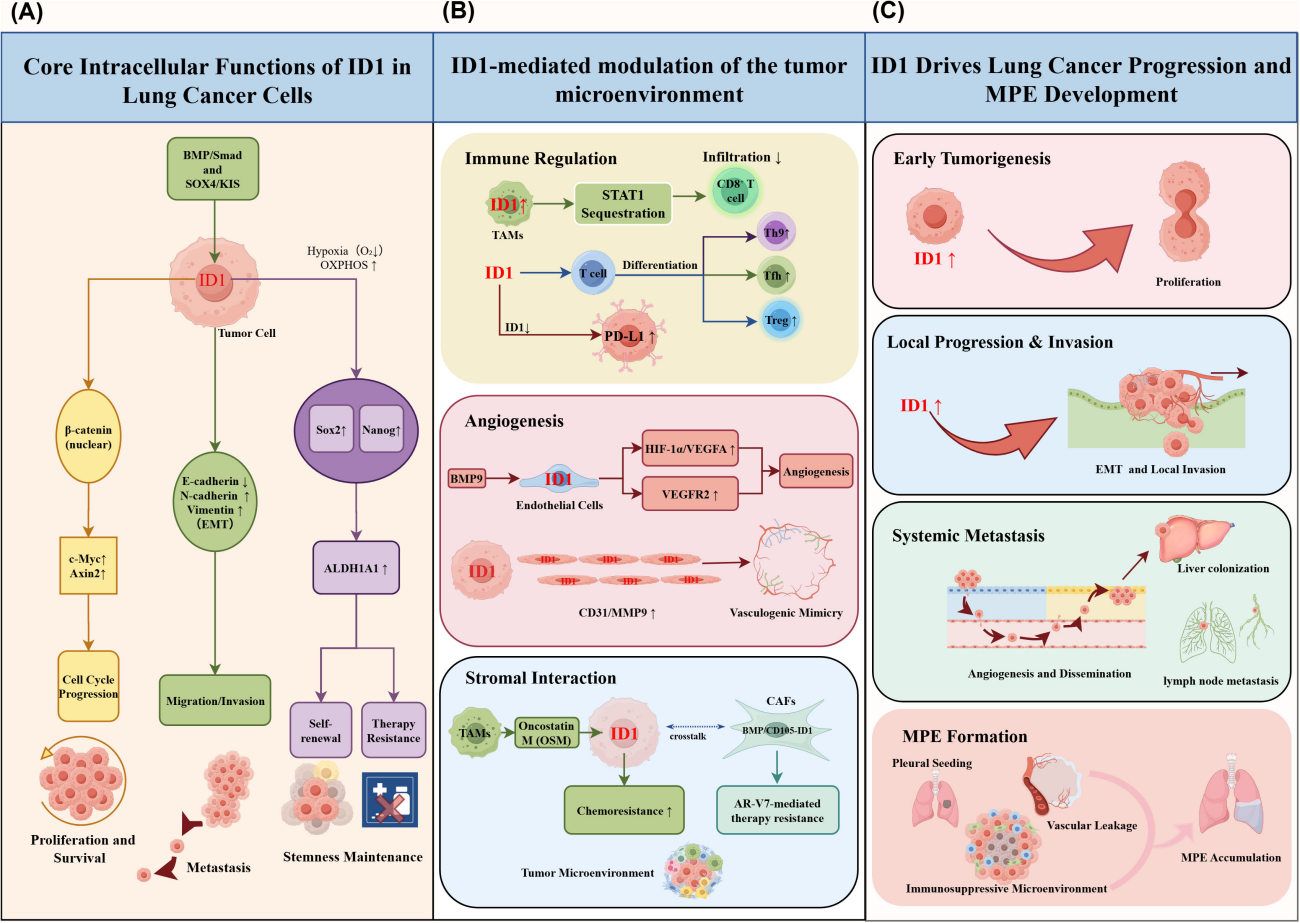
Schematic overview of the multifaceted roles of ID1 in lung cancer progression and its potential link to malignant pleural effusion. **(A)** Core intracellular functions. ID1, often upregulated by signals such as BMP/Smad and SOX4/KIS, drives three core malignant programs within lung cancer cells. First, it promotes proliferation and survival via β-catenin-mediated induction of c-Myc and Axin2, leading to cell cycle progression. Second, it mediates invasion and metastasis by inducing epithelial-mesenchymal transition (EMT; characterized by E-cadherin loss and N-cadherin/vimentin gain). Third, it maintains cancer stem cell properties, such as self-renewal and therapy resistance, by activating stemness-related transcription factors (e.g., Sox2/Nanog) and the marker ALDH1A1. Hypoxia and metabolic reprogramming (enhanced OXPHOS) further stabilize and reinforce this ID1-driven stemness. **(B)** Immune regulation: ID1 fosters immunosuppression through multiple mechanisms: (i) in tumor-associated macrophages (TAMs), ID1 sequesters STAT1 to limit CD8+ Tcell infiltration (validated in colorectal cancer, yet unconfirmed in lung cancer); (ii) in lung tumor cells, decreased ID1 elevates PD-L1 expression in KRAS-mutant lung adenocarcinoma (LUAD) (57); and (iii) in CD4+ T cells, ID1 promotes T helper 9 (Th9), follicular helper T cell (Tfh), and regulatory T cell (Treg) differentiation (findings derived from non-pulmonary immunological models; validation within lung cancer is still needed). Angiogenesis: ID1 drives angiogenesis through multiple mechanisms: in endothelial cells, ID1 upregulates HIF 1α/VEGFA (validated in hepatocellular carcinoma) and enhances VEGFR2 expression (established in endothelial progenitor cells), thereby promoting neovascularization; in tumor cells, ID1 elevates CD31/MMP 9 to induce vasculogenic mimicry (validated in breast and pancreatic cancers). Stromal Interaction: Tumor-associated macrophages (TAMs) secrete oncostatin M (OSM), which activates ID1 in cancer cells, contributing to chemoresistance (73). Cancer-associated fibroblasts (CAFs) harbor BMP/CD105-ID1 signaling, promoting AR-V7−mediated therapy resistance (75). Dashed lines indicate indirect crosstalk between cancer cells and CAFs; the specific role of ID1 in this crosstalk requires further investigation. Note: The OSM-ID1 chemoresistance axis is based on cholangiocarcinoma studies (73); BMP/CD105-ID1 signaling in CAFs promoting AR-V7−mediated therapy resistance is based on prostate cancer studies (75). The relevance of OSM signaling to lung cancer and the involvement of ID1 in CAF-tumor crosstalk remain to be established. Note: Several immunomodulatory and angiogenic mechanisms depicted here were originally established in other cancer types and require validation in lung cancer or the MPE setting. **(C)** Disease progression timeline. The functions of ID1 contribute sequentially to lung cancer progression and complications. Early Tumorigenesis: ID1-driven proliferation initiates tumor growth. Local Progression & Invasion: ID1-mediated EMT and local invasion facilitate tumor spread. Systemic Metastasis: ID1 promotes metastasis through two distinct routes: lymphatic spread to lymph nodes (via BMP/Smad) and hematogenous colonization of distant organs (e.g., liver) (via angiogenesis and stemness). MPE Formation: In advanced disease, ID1-associated processes—pleural seeding by invasive cells, vascular leakage from dysregulated angiogenesis, and establishment of an immunosuppressive microenvironment—are posited to mechanistically converge to drive the development of malignant pleural effusion (MPE). The proposed link between ID1 and MPE formation represents 'putative mechanisms' rather than validated pathways, and requires direct experimental confirmation. Solid arrows indicate activation or promotion. Created with Figdraw.

Thus, this review aims to systematically bridge this gap by synthesizing current knowledge on ID1's functions in lung cancer and critically evaluating the theoretical basis for its potential role in MPE pathogenesis. We propose that characterizing this link offers a promising avenue to elucidate understudied mechanisms of MPE formation. To support the detailed discussions that follow, **Figure 1** schematically summarizes the multifaceted roles of ID1 across the spectrum of lung cancer progression and its hypothesized involvement in MPE pathogenesis, serving as a conceptual framework for readers to contextualize the evidence assessment and testable model presented in Section 5.

## 2 The molecular structure and biological functions of ID1

### 2.1 Structural characteristics and basic functions of ID1 protein

ID1 protein belongs to the inhibitor of differentiation family (Inhibitor of Differentiation, ID). Its structural hallmark is the absence of a DNA-binding domain but the presence of a conserved helix-loop-helix (HLH) domain. Through this domain, ID1 forms heterodimers with basic helix-loop-helix (bHLH) transcription factors, thereby sequestering them from DNA binding and inhibiting their transcriptional activity. As a non-DNA-binding transcriptional regulator, ID1 functions as a pivotal regulator of cell differentiation and proliferation (24, 25).

Consistent with this broad role in cell fate control, ID1 is integral to maintaining stem cell pluripotency and an undifferentiated state during embryonic development—a function that underpins its subsequent role in cancer stem cells (CSCs) (26, 27). ID1 also exerts a direct influence on cell survival, serving as a key anti-apoptotic mediator in REIC/Dkk-3-induced apoptosis (28). Additionally, ID1 plays a regulatory role in angiogenesis; for instance, bone morphogenetic protein 2 (BMP2) induces ID1 expression, which promotes human trophoblast invasion and endothelial-like tube formation, highlighting ID1's importance in physiological and tumor-associated neovascularization (29).

The protein stability of ID1 is strictly regulated by the ubiquitin-proteasome system. Specifically, the E3 ubiquitin ligase TRIM21 promotes its degradation, whereas the deubiquitinase USP8 enhances its stability, collectively fine-tuning ID1's functional output (30–32). In summary, ID1 serves as a pivotal regulatory node in diverse processes—cell proliferation, differentiation, apoptosis, embryonic development and angiogenesis.

### 2.2 ID1 in cell fate determination: context dependent functions

ID1 is a pivotal regulator of cell fate, primarily functioning to maintain stemness and prevent differentiation across various biological contexts. This role stems from its fundamental capacity to inhibit the activity of bHLH transcription factors that drive cell-specific differentiation programs. Moreover, ID1 regulation of cell fate also involves its impact on the cell cycle and apoptosis. ID1 can promote cell cycle progression, prevent cells from entering the differentiation phase, and maintain a proliferative state (33).

In normal development and tissue homeostasis, ID1 preserves progenitor cell pools. For instance, in the epidermis, ID1 maintains basal progenitor cells by suppressing their differentiation into spinous layers through the inhibition of differentiation-inducing bHLH factors like TCF3/4 (34). This mechanism extends to the immune system, where ID1 promotes the differentiation of specific T-helper subsets, such as Th9 cells, by similarly repressing these transcriptional repressors (10). Thus, the same core mechanism—ID1 binding to TCF3/4—yields opposite outcomes: blocking differentiation in the epidermis but promoting it in Th9 cells. This context-dependent effect arises because TCF3/4 activate differentiation genes in the epidermis but repress IL-9 in T cells. ID1’s net effect is therefore not intrinsic but dictated by what its bHLH partners normally do in each cell type.

Beyond development, ID1's influence on cell fate extends to pathological tissue remodeling, such as pulmonary fibrosis. The expression of ID1 is rapidly induced by transforming growth factor-beta (TGF-β), a key regulator of fibrosis, in human lung fibroblasts and is highly expressed in (myo)fibroblasts within fibrotic lesions, positioning it as an early response gene in the fibrotic cascade (35). Supporting its pathological relevance, the anti-fibrotic agent cyclosporine A downregulates ID1 expression in human lung myofibroblasts (36). Intriguingly, *in vivo* functional studies reveal a complex role: genetic ablation of Id1 in mice exacerbates bleomycin-induced lung fibrosis, suggesting that ID1 may function to restrain excessive fibroblast activation in this specific setting (37). These seemingly contradictory findings can be explained by timing and partner switching. Early after TGF-β stimulation, ID1 binds classical bHLH factors that normally regulate fibroblast differentiation; by inhibiting these bHLH factors, ID1 promotes myofibroblast differentiation. Later, ID1 binds a non-bHLH partner, DRIL1 (ARID3A), which activates fibrotic genes; by sequestering DRIL1, ID1 limits excessive fibrosis. Thus, ID1 can either promote or limit fibrosis depending on when it acts and which partner it binds. The key features of these context-dependent outcomes are summarized in **Table 1**.

**Table 1:** Context-dependent outcomes of ID1 in normal physiology and pathological remodeling.

System	Upstream signal	ID1-binding partners	Normal function of partners	Net effect of ID1
Epidermis (34)	BMP	TCF3, TCF4,TCF12	Activate differentiation genes	Blocks differentiation, maintains stemness
Th9 differentiation (10)	IL-4 + TGF-β	TCF3, TCF4	Repress Il9 transcription	Promotes Th9 differentiation
Pulmonary fibrosis – early (35)	TGF-β	Classical bHLH factors	Regulate fibroblast differentiation program	Promotes myofibroblast differentiation
Pulmonary fibrosis – late (37)	TGF-β	DRIL1 (non-bHLH)	Enhance TGF-β-driven profibrotic gene expression	Limits excessive fibrosis

ID1 binds different partners in response to distinct upstream signals, leading to context-dependent outcomes in normal development, immunity, and tissue repair.

Together, these examples illustrate a central principle: ID1 has no intrinsic function of its own. Its net effect—whether blocking differentiation, promoting a specific lineage, or limiting tissue damage—is dictated by which partners it binds, when it acts, and what those partners normally do in each biological context.

## 3 The multifaceted roles of ID1 in lung cancer pathogenesis

### 3.1 Promotion of cell proliferation and survival

Elevated ID1 expression has been detected in lung cancer cell lines and clinical NSCLC specimens compared with normal counterparts (38). Clinical cohort analysis further supports the oncogenic functions of ID1 *in vivo*: in patients with locally advanced stage III-N2 NSCLC receiving definitive chemoradiotherapy, co-expression of ID1 and its closest family member ID3 is significantly associated with worse overall survival, with the prognostic effect being most pronounced in the T4N2 subgroup (39). This clinical correlation directly reflects the ability of ID1 to support tumor cell survival and proliferation to drive disease progression.

Functional validation in pre-clinical models corroborates these oncogenic roles: in EGFR T790M-mutant, osimertinib-resistant NSCLC cells, ID1 knockdown induces robust apoptosis and G1/G0 cell cycle arrest, directly confirming the indispensable roles of ID1 in sustaining NSCLC cell survival and proliferative capacity (40).

Mechanistically, ID1 promotes cell proliferation by activating the Akt signaling pathway (38) and also facilitates cell cycle progression via the SOX4-KIS-ID1-β-catenin axis in lung adenocarcinoma (LUAD) (41). Together, these findings establish ID1 as a key regulator linking multiple oncogenic signals to cell cycle progression and survival in lung cancer.

### 3.2 Mediation of invasion and metastasis

ID1 promotes invasion and metastasis in NSCLC through multiple mechanisms, most notably through the induction of epithelial-mesenchymal transition (EMT). EMT is a process whereby epithelial cells lose polarity and cell-cell adhesion and acquire migratory and invasive mesenchymal traits (42). In NSCLC, ID1 drives this program by repressing the epithelial marker E-cadherin and inducing mesenchymal markers such as N-cadherin and vimentin, thereby enhancing cellular motility and promoting liver colonization, as demonstrated in both *in vitro* and *in vivo* models (20).

Beyond lung cancer, studies across multiple tumor types have revealed that ID1 can function at distinct stages of the metastatic cascade, though the specific stage(s) involved vary by tumor type. During the initial dissemination phase, ID1 promotes EMT to enable cancer cell detachment and invasion, as demonstrated in gastric and cervical cancers, where ID1 acts downstream of BMP4/Smad and TBX3, respectively (43, 44). During the circulation and survival phase, ID1 uncouples EMT from apoptosis—a critical step for cell survival during dissemination—as shown in pancreatic cancer, where TGF-β induces both EMT and apoptosis in premalignant cells, whereas ID1 expression blocks the apoptotic arm while preserving the EMT program (45). During the final colonization phase, ID1 facilitates mesenchymal-to-epithelial transition (MET) to support metastatic outgrowth at distant organs, as demonstrated in breast cancer, where ID1 antagonizes Twist1 to promote MET and lung colonization (46). These observations do not imply that ID1 universally drives all three stages in every cancer; rather, they reveal a context-dependent flexibility: ID1 can be engaged at different metastatic steps depending on the tumor type, upstream signals, and microenvironmental pressures.

The pro-metastatic activity of ID1 in lung cancer is controlled by multiple upstream oncogenic pathways. The BMP pathway activates Smad1/5/9 phosphorylation, leading to increased ID1 transcription. Notably, NADK enhances NSCLC progression by interacting with Smurf1 to inhibit BMPR1A ubiquitination and degradation, thereby activating BMP signaling and upregulating ID1 expression, which correlates with increased lymph node metastasis and poorer prognosis (22). Additionally, the SOX4-KIS axis transcriptionally upregulates ID1, leading to sustained β-catenin activation and driving tumor cell proliferation, migration, and invasion in lung adenocarcinoma (41). Together, these findings establish ID1 as a key integrator of diverse upstream oncogenic signals that converge on metastatic programs (22, 41).

### 3.3 Maintenance of cancer stem cell properties

CSCs represent a subpopulation of tumor cells endowed with self-renewal capacity, multilineage differentiation potential, and intrinsic resistance to conventional therapies. They are widely regarded as the primary driver of tumor initiation, progression, distant metastasis, and therapeutic recurrence. ID1 has emerged as a key regulator of CSC stemness across diverse malignancies (1), yet the upstream signaling cascades and downstream functional mediators governing its activity exhibit marked tumor-type specificity.

In small-cell lung cancer (SCLC), the most direct evidence linking ID1 to lung CSC regulation has been established. ID1 is activated downstream of the BMP2/Smad signaling pathway, which is itself regulated by neuron-specific enolase (NSE). NSE downregulates neuroblastoma suppressor of tumorigenicity 1 (NBL1), alleviating its inhibitory effect on BMP2, and thus facilitates BMP2-mediated activation of Smad proteins and subsequent induction of ID1 expression. This cascade enhances the stem cell-like properties of SCLC cells, including increased proliferation, drug resistance, and spheroid formation (47). These findings provide a clear mechanistic framework for ID1-driven CSC regulation in lung cancer, albeit restricted to the SCLC subtype.

In LUAD, multi-omics subtyping identified CS3 as the subtype with the highest stemness score, suggesting enhanced invasive and metastatic potential (48). ID1 was also identified as a prognostic gene in the same study, with elevated expression correlating with unfavorable survival (48). However, whether ID1 specifically contributes to CSC properties in LUAD—particularly in the CS3 subtype—has not been experimentally validated.

Beyond lung cancer, extensive mechanistic studies in other malignancies have elucidated ID1-mediated CSC regulation. In breast cancer, the MYC-ZNF148-ID1/3 axis drives CSC properties through transcriptional derepression of ID1/3 (49), and Id1/3 promote CSC self-renewal through negative regulation of Robo1 (50). In glioma, ID1 promotes stemness through Tgm2/Akt signaling (51), whereas Cx43 restoration reverses this phenotype by suppressing c-Src-mediated ID1 expression (52), underscoring that ID1 inhibition can be achieved through distinct regulatory inputs. In hepatocellular carcinoma, macrophage-derived TWEAK induces ID1 expression via NF-κB signaling, driving malignant transformation of progenitor cells into CSCs (53). Additionally, mechanistic studies in cervical cancer models have revealed that elevated polyamine flux suppresses LSD1 activity, relieving epigenetic repression of the ID1 gene and thereby sustaining CSC stemness (54). These cross-cancer findings collectively demonstrate that ID1 is capable of driving CSC properties through diverse mechanisms—ranging from canonical signaling cascades to metabolic-epigenetic crosstalk—when activated in appropriate cellular contexts.

Collectively, these findings establish ID1 as a shared node for CSC maintenance across diverse malignancies, in which it promotes self-renewal, blocks differentiation, and confers therapy resistance. The upstream signals and downstream effectors engaged by ID1 vary considerably by tumor type—ranging from the NSE/NBL1/BMP2/Smad axis in SCLC to the MYC-ZNF148-ID1/3 cascade in breast cancer and TWEAK-NF-κB-ID1 signaling in hepatocellular carcinoma—highlighting its context-dependent yet versatile nature. In LUAD, although NADK/BMP (22) and SOX4-KIS (41) pathways have been functionally linked to ID1-driven proliferation and metastasis, direct evidence for ID1-mediated CSC regulation in this subtype remains limited. Future investigations should prioritize functional assays in LUAD models to determine whether ID1 directly sustains CSC properties and to define the upstream signals that activate ID1 in this context. This question is of particular relevance, as ID1-driven CSC programs likely cooperate with proliferative and metastatic signaling networks rather than functioning in isolation. By simultaneously coordinating self-renewal, invasive capacity, and survival, ID1 orchestrates a self-amplifying malignant circuit, rendering it a common therapeutic vulnerability across cancers—though precise patient stratification by tumor subtype and molecular context will be essential for effective targeting.

### 3.4 Regulatory network of ID1 expression in lung cancer cells

The expression of ID1 in lung cancer cells is governed by a multilayered regulatory network involving transcriptional control, post-translational modulation, and extracellular signals.

At the transcriptional level, PGC1α and FOXA1 cooperate to activate ID1 expression. TGFβ1 suppresses FOXA1 and PGC1α, leading to decreased ID1 expression (55). Additionally, the transcription factor SOX4 upregulates KIS, which in turn enhances ID1 expression (41).

ID1 protein stability is additionally controlled by the ubiquitin-proteasome system. In KRAS-mutant LUAD, MEK inhibition downregulates ID1 via proteasome-mediated degradation (23).

The BMP signaling pathway serves as a major activator of ID1 transcription in lung cancer. NADK stabilizes BMP receptors, enhancing BMP signaling and driving ID1 upregulation (22). RGMb, a modulator of BMP signaling, has been shown to influence BMP2-induced ID1 and ID2 mRNA expression (56).

Collectively, ID1 expression in lung cancer is controlled by a network of transcriptional regulators (PGC1α/FOXA1, SOX4/KIS), post-translational mechanisms (proteasome-mediated degradation), and extracellular signals (BMP, TGFβ). This regulatory complexity positions ID1 as a convergence point for diverse oncogenic and stromal signals that influence tumor behavior.

## 4 Regulatory functions of ID1 in the tumor microenvironment

### 4.1 Regulation of immune cell function

Within the tumor microenvironment, ID1 modulates immune cell function through cell-type-specific mechanisms, with implications for tumor immune evasion and therapy resistance.

In KRAS-mutant LUAD, Id1 silencing relieves immunosuppression within the tumor microenvironment, leading to increased CD8^+^ T cell infiltration, and concurrently upregulates PD-L1 expression on tumor cells. Concurrent PD-1 blockade synergistically impairs tumor growth (57). Furthermore, MEK inhibition downregulates ID1 via proteasome-mediated degradation, similarly sensitizing KRAS-mutant tumors to PD-1/PD-L1 blockade (23). These observations suggest that ID1 contributes to an immunosuppressive TME in lung cancer by restricting intratumoral infiltration of CD8^+^ effector T cells, and that its inhibition creates a permissive context for immune checkpoint blockade.

Mechanistic insights from other malignancies have elucidated how ID1 orchestrates immunosuppression. In colorectal cancer, ID1 expression in tumor-associated macrophages (TAMs) correlates with poor prognosis; mechanistically, ID1 sequesters STAT1 in the cytoplasm, suppressing STAT1-target genes including CCL4 and SerpinB2, thereby limiting CD8^+^ T cell recruitment and maintaining cancer stemness (58). In hepatocellular carcinoma, the ID1/Myc axis upregulates PD-L1 expression and recruits polymorphonuclear myeloid-derived suppressor cells (PMN-MDSCs), driving immune evasion and chemotherapy resistance (59).

Beyond the tumor microenvironment, ID1 also shapes adaptive immunity by regulating T helper cell differentiation. In CD4^+^ T cells, ID1 promotes Th9 differentiation by relieving Tcf3/Tcf4-mediated repression of IL-9 (10) and augments follicular helper T (Tfh) cell development and function (60). In addition, ID1 lowers the activation threshold of naïve CD4^+^ T cells, yet favors the differentiation of immunosuppressive regulatory T cells (Tregs) under conditions of limited co-stimulation (61, 62). In the B cell lineage, BMP-6/Smad signaling induces ID1 upregulation to act as an intrinsic brake that restrains early B progenitor proliferation and differentiation (63, 64). Collectively, these observations derived from non-lung immunological models highlight the highly context-dependent nature of ID1 function across diverse immune cell subsets.

Collectively, ID1 has been shown to foster immunosuppression through diverse cell-type-specific mechanisms across different cancer types, ranging from TAM-mediated immune suppression and CD8^+^ T cell exclusion to Treg differentiation and MDSC recruitment. In lung cancer specifically, direct evidence supports ID1‘s role in CD8^+^ T cell exclusion and PD-L1 regulation, and therapeutic targeting of ID1 has been shown to sensitize tumors to PD-1/PD-L1 blockade. However, direct mechanistic validation of ID1-driven immunosuppression in the lung TME—particularly in TAMs and other myeloid compartments—remains limited compared to other cancer types. The convergence of these ID1-controlled immunosuppressive cascades across diverse malignancies nonetheless establishes ID1 as a valuable therapeutic candidate to remodel the suppressive tumor microenvironment and overcome immunotherapy resistance, especially when combined with MEK inhibitors or immune checkpoint blockade agents.

### 4.2 Modulation of tumor angiogenesis

ID1 plays a critical role in tumor angiogenesis by regulating endothelial cell function and angiogenic signaling pathways, though the mechanisms and the strength of evidence vary considerably by cancer type.

In lung cancer, direct evidence for ID1-driven angiogenesis is limited. In SCLC, chemotherapy has been reported to upregulate ID1 in stalk-like endothelial cells, enhancing their angiogenic activity (65). Additionally, sulfated galactoglucan derivatives that block BMP/Smad/ID1 signaling have been shown to reduce VEGF expression and suppress lung tumor growth, suggesting that the BMP/ID1 axis may be therapeutically targetable in lung cancer (66).

Mechanistic insights from other cancer types and model systems have revealed key pathways through which ID1 promotes angiogenesis. In endothelial progenitor cells (EPCs), ID1 acts as a transcriptional modulator of VEGFR2 by relieving E2-2-mediated repression of the VEGFR2 promoter, thereby enhancing VEGFR2 expression and signaling capacity (67–69). In hepatocellular carcinoma, the BMP9 pathway induces ID1 expression, which upregulates HIF-1α and VEGFA, promoting VEGF secretion and neovascularization (70). ID1 also facilitates vasculogenic mimicry—a process by which tumor cells form vessel-like networks—in breast and pancreatic cancers through the expression of pro-angiogenic proteins including VEGF, CD31, and MMP9 (4).

In glioblastoma, ID1 drives VEGF-independent angiogenesis and resistance to anti-VEGF therapy through two complementary mechanisms. First, ID1 upregulates endothelin-1 (EDN1), which promotes endothelial sprouting via EDNRB and exhibits stronger pro-angiogenic activity than VEGF (71). Second, ID1 induces activin A secretion, which through the Smad3/Slug axis triggers endothelial-to-mesenchymal transition (EndoMT) and endothelial cell apoptosis, leading to vascular abnormalities—including reduced vessel density, increased permeability, and dispersed hypoxia—that confer resistance to bevacizumab (72). These findings illustrate the multifaceted nature of ID1-mediated angiogenesis and suggest that combined targeting of ID1 downstream effectors may be required to overcome anti-VEGF resistance.

Thus, ID1 integrates multiple angiogenic axes—VEGFR2 modulation, BMP9/VEGFA signaling, vasculogenic mimicry, EDN1-driven sprouting, and activin A-mediated vascular remodeling—across diverse cancers. In lung cancer, however, the landscape is less comprehensively characterized. Direct evidence is largely confined to chemotherapy-induced ID1 upregulation in endothelial cells and pro-angiogenic signaling downstream of activated BMP-ID1 cascades. Whether ID1 orchestrates additional angiogenic programs in the lung TME—including VEGFR2 modulation, activation of BMP9/VEGFA signaling, or induction of vascular remodeling—remains largely unexplored. Future studies are warranted to systematically dissect ID1-mediated angiogenic mechanisms in lung cancer models and to determine whether the mechanistic paradigms established in other malignancies are generalizable to the lung context.

### 4.3 Interaction with tumor stromal cells

Within the tumor microenvironment, stromal populations—including cancer-associated fibroblasts (CAFs), TAMs, and myeloid-derived suppressor cells (MDSCs)—critically drive tumor progression via ECM remodeling, angiogenesis, and reciprocal tumor-stroma crosstalk. ID1 modulates the function of these stromal components, though supporting experimental evidence varies considerably across cell subsets and cancer types.

The role of ID1 in TAMs and MDSCs has been primarily characterized in non-lung cancer models. In cholangiocarcinoma, TAM-secreted oncostatin M activates the YAP-ID1 axis in cancer cells, driving proliferation and chemoresistance (73). In melanoma models, tumor-derived TGF-β induces ID1 upregulation in hematopoietic myeloid cells, suppressing IRF8, blocking dendritic cell maturation, and skewing myeloid precursors toward an immunosuppressive MDSC phenotype, thereby inhibiting CD8^+^ T cell responses and promoting tumor growth and metastasis (74). While these findings establish ID1 as a regulator of myeloid compartment function, direct validation in the lung TME is still lacking.

Beyond myeloid cells, ID1 also intersects with CAF biology in other tumor types. In CAFs, ID1 has been directly linked to stromal-tumor crosstalk in prostate cancer. ARSI treatment induces IL-6 secretion, which activates CD105/BMP signaling to upregulate ID1; elevated ID1 further drives RBM38 expression, promoting the generation of the constitutively active AR-V7 splice variant and driving therapy resistance (75). In NSCLC, CAFs contribute to immune evasion through multiple mechanisms—including impairment of dendritic cell function, altered macrophage polarization, reduced cytotoxic T cell infiltration, and direct inhibition of T cell proliferation via COX-2/PD-L1 pathways (76, 77). Although direct evidence linking ID1 to these CAF functions in lung cancer is currently lacking, the pathways implicated in NSCLC CAF-mediated immunosuppression intersect with known ID1 regulatory networks in other contexts—an observation that merits further investigation.

Collectively, ID1 acts as a conserved stromal modulator across diverse cancers, coordinating TAM-driven chemoresistance, aberrant MDSC differentiation, and CAF-mediated treatment failure. Mechanistic insights into ID1-stroma crosstalk are mostly extrapolated from non-lung malignancies, with scarce direct evidence in the lung TME. Further research is required to clarify whether ID1 exerts comparable regulatory effects on lung CAFs and myeloid cells, and whether stromal ID1 targeting can improve therapeutic responses. Even so, its multi-cancer role in shaping protumor stromal niches marks ID1 as a promising candidate to reverse microenvironment-mediated immune suppression.

### 4.4 Integration of ID1 within TME signaling networks

The preceding sections have delineated how ID1 functions across multiple cellular compartments—tumor cells, immune cells, endothelial cells, and stromal fibroblasts—to drive malignant progression. Here, we synthesize these observations into an integrated view of ID1 as a signaling hub that converges oncogenic pathways with microenvironmental cues.

ID1 integrates signals from at least three interconnected dimensions. First, within tumor cells, ID1 promotes proliferation and survival through Akt and β-catenin signaling, drives invasion via EMT programs, and sustains cancer stem cell properties (Sections 3.1–3.3). Second, within the immune microenvironment, ID1 orchestrates immunosuppression by modulating TAM function, restricting CD8^+^ T cell infiltration, and modulating T helper cell fate (Section 4.1). Third, within the vascular and stromal compartments, ID1 regulates angiogenesis through VEGFR2 and BMP9/VEGFA signaling, and has been linked to CAF-mediated therapy resistance through its role as a downstream effector of inflammatory signals (Sections 4.2–4.3).

Critically, these compartments are not isolated but communicate through ID1-dependent crosstalk. TAM-derived oncostatin M activates ID1 in tumor cells to drive chemoresistance (73); CAF-derived IL-6 signals through BMP/CD105 to induce ID1 and promote therapy resistance (75); and hypoxia stabilizes ID1 to enhance tumor cell survival and invasion (30). Thus, ID1 serves as a convergence point for signals emanating from diverse cellular sources within the TME.

This integrative capacity has therapeutic implications. Rather than targeting individual downstream pathways, disrupting the ID1 axis may simultaneously impair tumor cell fitness, relieve immunosuppression, and normalize the stromal and vascular microenvironment. In KRAS-mutant LUAD, MEK inhibition downregulates ID1 and sensitizes tumors to immune checkpoint blockade, illustrating how targeting an upstream regulator of ID1 can produce multi-compartment effects (23). Collectively, these observations position ID1 as a rational node for therapeutic intervention, though the context-dependent nature of its functions—varying by tumor type, cell compartment, and microenvironmental context—underscores the need for precise patient stratification and combination strategies.

## 5 Hypothesis: ID1 as a putative contributor to malignant pleural effusion formation

### 5.1 Pathophysiological basis of malignant pleural effusion

Malignant pleural effusion (MPE) is a common and debilitating complication of advanced lung cancer. It arises from a complex interplay between tumor cells and host factors, leading to the accumulation of exudative fluid in the pleural space (78, 79). The orchestration of this process is defined by three interrelated pathophysiological pillars, which have been conceptualized as a vicious loop of tumor-host interactions (80).

Tumor cell invasion and seeding of the pleura are essential initiating events (81). Malignant cells must detach from the primary tumor, migrate into the pleural cavity, and attach to the mesothelial lining—a process requiring the acquisition of invasive and adhesive properties, traditionally associated with cellular programs such as EMT. Notably, tumor cells isolated directly from MPEs exhibit a mesenchymal phenotype and cancer stem cell properties (82). Whether this mesenchymal state represents a prerequisite for pleural entry or a secondary adaptation to the TGF-β-rich pleural environment remains a subject of active debate; nevertheless, such features are functionally consistent with the enhanced survival and seeding capabilities required for pleural metastasis. Once established, pleural tumor deposits secrete a variety of vasoactive and immunomodulatory factors that remodel the local microenvironment.

Vascular hyperpermeability and pathological angiogenesis drive fluid accumulation. Increased permeability of pleural vessels allows plasma to leak into the pleural space, while new blood vessel formation sustains this process. Multiple growth factors and cytokines contribute to these vascular changes, with members of the VEGF family playing a central role (83).

The MPE microenvironment is profoundly immunosuppressive, enabling tumor cells to evade host immunity. Compared with peripheral blood, MPE exhibits a markedly different immune landscape: CD4^+^ T cells and Tregs are enriched, while cytotoxic CD8^+^ T cells and natural killer (NK) cells are reduced in number and function. In addition, macrophages display an M2-polarized phenotype that promotes tumor growth and angiogenesis. Collectively, these compositional and functional alterations favor tumor survival and progression (84, 85).

Integrating these diverse pathological facets of MPE, we note that the transcriptional regulator ID1 exhibits functional properties that conceptually converge on each of these three pathophysiological pillars. In various other cancer types, ID1 has been demonstrated to promote tumor cell invasion and metastasis, enhance angiogenic signaling, and orchestrate immunosuppressive networks. However, it is critical to emphasize that direct experimental or clinical evidence linking ID1 specifically to MPE is currently entirely absent. Therefore, the following sections do not describe established MPE mechanisms, but rather outline a conceptual framework—based on indirect analogies from other malignancies—to explore whether ID1 could theoretically contribute to pleural pathophysiology. We explicitly distinguish these indirect inferences from the testable predictions that will be proposed in Section 5.4. This distinction serves as a necessary safeguard against overinterpretation of the current evidence.

### 5.2 Vascular dysfunction and pleural seeding: conceptual parallels and testable entry points

We begin by examining two pathophysiological pillars of MPE—vascular dysfunction and tumor seeding—and ask whether ID1's known functions in other contexts might hypothetically intersect with these processes. It must be stressed at the outset that the following mechanistic links are speculative and await direct validation in the pleural space.

#### 5.2.1 ID1 as a putative regulator of vascular hyperpermeability in MPE

VEGF-driven leakiness of pleural blood vessels is a key step in MPE formation (15, 86). ID1 can enhance VEGF signaling in two well-established ways. First, it boosts VEGFR2 expression in endothelial cells by removing a natural brake called E2-2 (68, 69). Second, it acts downstream of BMP9 to increase HIF-1α and VEGFA, promoting abnormal blood vessel growth (70).

These findings are derived from liver cancer and endothelial progenitor cell studies, but the VEGFR2–ID1 pathway is likely active in many vascular beds. It is therefore a testable hypothesis that ID1 activity in pleural endothelial cells or tumor cells contributes to the excessive leakiness driving MPE.

In a mouse model of lung cancer implanted into the chest cavity, different VEGF family members had distinct effects: VEGF-A caused bloody pleural effusion, whereas VEGF-C and VEGF-D promoted lymph node metastasis and pleural tumor spread (87). ID1 is known to promote angiogenesis and vasculogenic mimicry in other cancers, partly through upregulating pro-angiogenic and pro-inflammatory factors such as VEGF-A, IL-8, and MMP9 (4). Whether ID1 might similarly influence VEGF-C or VEGF-D expression in the pleural space is unknown, but this functional overlap raises a testable hypothesis for future investigation.

#### 5.2.2 ID1 in tumor seeding: EMT and mesothelial reprogramming

Successful pleural seeding requires tumor cells to penetrate the mesothelial barrier and establish metastatic deposits. ID1 promotes EMT in lung cancer models, thereby enhancing cancer cell invasion and metastasis (20). This pro-invasive capacity directly supports the invasive and adhesive capabilities needed for pleural dissemination, raising the possibility that ID1 contributes to tumor cell entry into the pleural space.

Beyond its cell-autonomous effects, ID1 may also modulate the pleural mesothelium itself. Pleural mesothelial cells (PMCs) undergo TGF-β-induced mesothelial-to-mesenchymal transition (MesoMT), adopting a myofibroblast-like phenotype that disrupts mesothelial integrity and facilitates tumor adhesion and invasion (88, 89). ID1 is a canonical downstream effector of TGF-β. In peritoneal mesothelial cells, ID1 mediates TGF-β1-induced VEGF-A production (90), indicating that ID1 can drive mesothelial responses in serosal cavities. It is therefore plausible—though unproven—that ID1 similarly promotes MesoMT in the pleura, thereby priming the mesothelial "soil" for colonization.

Supporting the relevance of tumor-stromal crosstalk, CAF-derived extracellular vesicles can promote lung adenocarcinoma metastasis by suppressing the ID1–IGFBP3 axis (91). Interestingly, this suggests that ID1 downregulation may enhance metastasis, which seems to contrast with ID1's generally pro-tumorigenic role. This apparent contradiction highlights that ID1's function is context-dependent and may vary at different stages of the metastatic cascade. For example, the optimal efficiency of metastasis might require dynamic regulation of ID1 during tumor cell detachment, survival in pleural fluid, or attachment to the mesothelium. Such dual (biphasic) roles of transcription factors, where their effects can differ depending on cellular context or timing, are increasingly recognized in the regulation of cellular plasticity. In the pleural space, understanding whether ID1 promotes or suppresses tumor seeding requires MPE-specific functional studies.

Direct evidence linking ID1 to pleural seeding or mesothelial reprogramming in MPE is currently absent, and each of these proposed mechanisms awaits experimental validation.

### 5.3 Immune regulation by ID1: a conceptual framework for the MPE immunosuppressive niche

We next consider whether ID1 could hypothetically shape the unique immune landscape of MPE. The following discussion is therefore framed as a conceptual alignment between ID1's immunomodulatory functions established in other cancers and the known immunosuppressive features of MPE, rather than as established MPE biology.

As outlined in Section 5.1, the MPE microenvironment is profoundly immunosuppressive. ID1 is known to orchestrate immunosuppressive programs across multiple cancer types, raising the possibility that it may similarly modulate the immune landscape within the pleural space.

In colorectal cancer, ID1 expression in TAMs restricts CD8^+^ T cell infiltration by sequestering STAT1 in the cytoplasm and reducing CCL4 production (58). This mechanism may be relevant to MPE, where CD8^+^ T cells are scarce and immunosuppressive TAMs dominate, although direct evidence in MPE-resident macrophages is currently lacking.

In adaptive immunity, ID1 significantly influences T cell differentiation. Within the murine CD4^+^ subset, ID1 promotes Th9 differentiation by inhibiting the transcriptional repressors Tcf3 and Tcf4 (10). Correspondingly, pleural mesothelial cells in MPE have been shown to recruit Th9 cells via CCL20 secretion, contributing to the characteristic Th9 enrichment observed in the effusion (92). These findings suggest two complementary pathways through which ID1 could orchestrate Th9 accumulation in MPE: indirectly, by modulating mesothelial chemokine production, or directly, by driving Th9 differentiation within the pleural niche. Neither mechanism has yet been experimentally validated.

Additionally, in murine T cells, ID1 promotes Treg development under conditions of low co-stimulation (61). In MPE, Tregs are actively recruited via CCL22 (93), but whether ID1 expression in pleural T cells contributes to this recruitment or functional polarization remains unexplored.

Beyond adaptive immunity, ID1 may also influence innate immune responses. Elevated ID1/ID3 levels in B-cell acute lymphoblastic leukemia correlate with myeloid skewing and a cytokine milieu that enhances neutrophil activation and immune suppression (8). Similarly, MPE fluid can induce pro-tumor neutrophil phenotypes, such as enhanced survival and the release of neutrophil extracellular traps (NETs) (94), suggesting a potential role for ID1 in shaping innate immunity within the pleural space, though direct evidence is absent.

Beyond its active role in promoting immunosuppression, ID1 may also interfere with endogenous anti-tumor responses. For instance, the presence of eosinophils in MPE—driven in part by IL-5—has been paradoxically associated with favorable outcomes (95, 96). While a direct regulatory link between ID1 and eosinophil biology remains to be established, ID1's established capacity to skew myeloid differentiation toward suppressive lineages suggests that high ID1 activity might indirectly constrain the development or recruitment of such beneficial immune subsets. This 'myeloid bias' represents another layer of the complex immune landscape potentially orchestrated by ID1.

Collectively, these observations position ID1 as a putative orchestrator of immune exclusion and polarization in MPE. However, direct experimental validation in pleural-specific contexts is entirely lacking, highlighting critical avenues for future investigation.

### 5.4 Evidence gaps and a proposed experimental roadmap

The established functions of ID1—including promoting vascular leakage, enhancing tumor invasion, and regulating immune suppression—closely align with the core pathophysiological processes of MPE, providing a strong mechanistic rationale for its potential involvement. However, none of these activities has been directly demonstrated within the pleural microenvironment, and the role of ID1 in MPE remains unsupported by direct experimental evidence.

Moving from hypothesis to proof requires addressing two fundamental questions. First, it is necessary to determine whether ID1 is expressed in the pleural environment and, if so, in which cell types. Single-cell transcriptomic or multiplex immunohistochemical analysis of freshly isolated MPE samples from well-annotated patient cohorts could reveal the cellular distribution of ID1—tumor cells, macrophages, pleural mesothelial cells, or endothelial cells—and assess whether its expression correlates with effusion volume, VEGF concentration, immune cell composition, or clinical outcomes. Among these candidate compartments, tumor cells represent the most plausible primary source of ID1-mediated MPE pathogenesis, given the well-established role of ID1 in promoting lung cancer cell proliferation, survival, EMT, and stemness (Sections 3.1–3.3). Nevertheless, given that ID1 also modulates immune cell function, endothelial cell biology, and mesothelial responses in other contexts, its potential contribution from non-tumor compartments should not be ruled out. A tiered experimental approach could therefore be envisioned: initial studies using tumor cell–specific Id1 deletion would assess whether tumor-intrinsic ID1 contributes to pleural fluid accumulation. If tumor cell deletion yields only partial effects, subsequent cell type–specific knockout strategies—targeting myeloid cells, endothelial cells, or mesothelial cells—could systematically dissect the contribution of each stromal compartment. Additionally, pharmacological inhibition of ID1—which would target both tumor and stromal compartments simultaneously—could provide a complementary approach to evaluate the integrated effect of ID1 across multiple cell types.

Second, it is essential to establish whether ID1 actively drives MPE formation *in vivo*. Using established murine MPE models—such as orthotopic lung cancer models with pleural metastasis established by intrathoracic injection of syngeneic tumor cells (e.g., LLC cells in immunocompetent mice)—cell type–specific genetic manipulation or pharmacological inhibition of ID1 could be employed to evaluate its impact on effusion accumulation, vascular permeability, tumor dissemination, and immune landscape remodeling.

This experimental framework yields clear, falsifiable predictions. If ID1 actively drives MPE formation, its genetic or pharmacological inhibition should reduce pleural fluid volume, decrease vascular permeability, limit tumor cell seeding, and/or restore CD8^+^ T cell-mediated immunity within the pleural space. Conversely, if ID1 is dispensable for MPE, its manipulation would produce no significant changes in these endpoints—an equally informative result that would redirect attention to other molecular drivers of pleural pathogenesis.

Importantly, the net effect of ID1 in MPE may depend on the cellular compartment (tumor cells vs. immune cells vs. mesothelial cells) and the stage of pleural disease (early seeding vs. established effusion). This underscores the need for cell-type-specific and temporally controlled interventions in future studies.

Until such studies are conducted, the role of ID1 in MPE should be regarded as a testable hypothesis rather than an established mechanism. Addressing these questions will move research from mechanistic inference to causal evidence, providing a foundation for evaluating ID1 as a potential therapeutic target in MPE.

## 6 Clinical translation of ID1: from paradoxical functions to precision strategies

### 6.1 ID1 as a biomarker: a dualistic portrait in prognosis and predictive value

Cumulative data delineating ID1’s molecular functions establish it as a key mediator of lung cancer progression. In this section, we integrate its clinical utility as a biomarker, noting marked divergence between prognostic (long-term outcome stratification) and predictive (treatment response forecasting) applications.

As a prognostic biomarker, the evidence is relatively robust. High ID1 protein levels, detected by immunohistochemistry, serve as an independent predictor of shorter disease-free and overall survival in NSCLC patients, particularly in those with adenocarcinoma histology (97). This association has been observed across distinct lung cancer subtypes: co-expression of ID1 and ID3 correlates with worse outcomes in stage III-N2 NSCLC patients treated with definitive chemoradiotherapy (39), and in SCLC, ID1 driven by the NSE/BMP2/Smad axis defines an aggressive subset with stem-cell-like properties and inferior prognosis (47). Loss of ID1 repressors, such as miR-381 and miR-29b, further reinforces the association between high ID1 activity and poor outcomes (98, 99). Mechanistically, these prognostic associations are supported by ID1‘s established roles in promoting proliferation, EMT, metastasis, and stemness—including lymph node metastasis through the NADK/BMP/ID1 axis (22) (Sections 3.1–3.3).

However, ID1's value as a predictive biomarker—guiding specific therapy choices—is nuanced and context-dependent, exemplifying its functional duality. This is most evident in ID1‘s opposing effects on EGFR-TKIs, where the same molecular regulator can drive resistance to third-generation inhibitors (osimertinib) yet enhance sensitivity to first-generation inhibitors (gefitinib).

These seemingly contradictory findings are not irreconcilable; rather, they reflect a deeper determinant: the cellular EMT state. In cells that have already undergone EMT and acquired a mesenchymal phenotype—such as gefitinib-resistant PC9/GR and H1975 cells—ID1 is endogenously upregulated and functions as a "survival switch," stabilizing the mesenchymal state through BMP2/Smad/ID1-driven EMT and thereby promoting resistance (40, 100). In epithelial-like cells that have not undergone EMT, however, forced ID1 overexpression activates a distinct pathway: it maintains Akt activity and upregulates cFLIP, which dissociates the caspase-8-RIP1 complex and shifts cell death from apoptosis to RIP3/MLKL-dependent necroptosis, thereby enhancing gefitinib sensitivity (101). Thus, ID1 does not possess an intrinsic pro-resistance or pro-sensitivity function; rather, its net effect is dictated by the EMT status of the cell and the level of ID1 expression—stabilizing mesenchymal traits under endogenous upregulation in mesenchymal cells, yet shifting cell death modality toward necroptosis upon forced overexpression in epithelial-like cells. Within the EGFR-TKI setting, ID1 can therefore be classified as harmful in mesenchymal cells where it sustains EMT-driven resistance, and beneficial in epithelial-like cells where forced ID1 overexpression triggers necroptosis.

Beyond EMT status, the treatment context further shapes ID1‘s predictive value. ID1's effect on drug response is not determined by a drug's target class, but by the specific cellular pathway it engages. This is most clearly illustrated by EGFR-TKIs: ID1 enhances sensitivity to first-generation inhibitors (gefitinib, erlotinib) through necroptosis, yet drives resistance to the third-generation inhibitor osimertinib through EMT (40, 100, 101). The direction of ID1's effect thus depends on whether the treatment context allows ID1 to activate a death pathway (as in gefitinib-treated epithelial-like cells) or reinforces a survival pathway (as in osimertinib-resistant mesenchymal-like cells in which EMT-driven resistance has been established). Beyond EGFR-TKIs, similar context-dependence has been observed with sorafenib, where ID1 overexpression suppresses EMT to enhance efficacy despite sorafenib-induced ID1 downregulation (102), and with MEK inhibitors such as trametinib, where ID1 downregulation remodels the immune microenvironment to sensitize tumors to PD-1/PD-L1 blockade (23). In the immunotherapy setting, ID1 blockade has been shown to enhance the antitumor efficacy of PD-1 inhibitors in KRAS-mutant LUAD models, further supporting its context-dependent role in treatment response (57). These observations suggest that ID1's predictive value is broadly shaped by the interplay between drug mechanism and cellular state rather than by drug class alone.

Considered together, these contextual determinants—EMT status and treatment context—explain why ID1 is not a simple "high-is-bad" biomarker. Its prognostic value is broadly consistent across NSCLC subtypes, but its predictive value is highly context-dependent. This duality underscores that clinical interpretation of ID1 expression requires precise stratification by EMT status and therapeutic setting—a prerequisite for translating ID1 into clinical decision-making.

### 6.2 ID1 in therapy response and resistance: a dualistic target and the challenge of heterogeneity

This duality extends from its role as a biomarker to its direct impact on therapy. ID1 has emerged as a pivotal molecular determinant of therapeutic efficacy, exhibiting a complex and context-dependent influence on drug sensitivity and resistance across cancer types, including lung cancer. Critically, this context-dependency manifests across three interrelated dimensions: bidirectional effects within tumor cells depending on the specific therapeutic agent, compartment-specific functions across tumor, stromal, and immune cells, and differential roles across molecular subtypes and therapeutic backgrounds. This dualistic role positions ID1 not only as a biomarker of treatment response but also as a challenging yet promising therapeutic target.

First, within tumor cells, ID1 exhibits striking bidirectional effects depending on the specific therapeutic agent—most notably its opposing impact on EGFR-TKIs, where it enhances sensitivity to first-generation inhibitors (gefitinib) yet drives resistance to the third-generation inhibitor osimertinib (40, 100, 101). A similar paradox is observed with sorafenib, where ectopic ID1 expression can enhance efficacy in NSCLC by suppressing EMT (102). Collectively, these findings establish that ID1 functions as a contextual determinant rather than a binary switch; its clinical outcome is the integrated product of the drug's mechanism and the cell's internal signaling state.

Second, and critically for understanding therapeutic resistance, ID1's functions are highly compartment-specific, operating through distinct mechanisms in tumor cells versus stromal and immune cells within the TME. This compartment-specificity directly shapes the net therapeutic outcome. In the tumor cell compartment, ID1 promotes resistance to chemotherapy and targeted agents through cell-autonomous mechanisms. For instance, in KRAS-mutant LUAD, ID1 contributes to acquired resistance to the MEK inhibitor trametinib, while its downregulation concurrently sensitizes tumors to PD-1/PD-L1 immune checkpoint blockade by remodeling the immunosuppressive TME (23). In ovarian cancer, ID1 confers cisplatin and paclitaxel resistance by activating a STAT3/ATF6-mediated autophagic survival pathway (103), and in ovarian cancer stem cells, autophagy-mediated ID1 degradation reverses chemoresistance through a mechanism specific to the stem cell compartment (104). In paclitaxel-resistant lung cancer cells, ID1 is among the upregulated resistance inducers, and its downregulation correlates with restored drug sensitivity (105).

In contrast, within the immune and stromal compartments, ID1 exerts pro-tumorigenic and resistance-promoting effects through distinct mechanisms that often operate independently of its expression in cancer cells. In colorectal cancer, high ID1 expression in TAMs sustains cancer stemness and limits CD8^+^ T cell infiltration by sequestering STAT1 in the cytoplasm, thereby suppressing the transcription of key chemokines such as CCL4 (58). This macrophage-specific ID1 function creates an immunosuppressive niche that indirectly fosters therapy resistance. Furthermore, in cholangiocarcinoma, TAM-secreted OSM activates the YAP-ID1 axis in cancer cells, driving proliferation and chemoresistance—a clear example of cross-compartment signaling where ID1 in one cell type (TAM) influences drug response in another (tumor cell) (73). Within the endothelial compartment, chemotherapy has been shown to upregulate ID1 specifically in stalk-like endothelial cells in SCLC, enhancing their angiogenic activity and potentially contributing to a pro-tumorigenic vascular niche that supports tumor survival under therapeutic stress (65).

Third, the functional heterogeneity of ID1 extends beyond compartment-specificity to encompass differential roles across cancer types and molecular subtypes, as well as context-dependent regulation at the post-translational level. In B-cell acute lymphoblastic leukemia (B-ALL), elevated ID1/ID3 expression correlates with an altered myeloid compartment and a cytokine milieu that promotes neutrophil activation and immune suppression, contrasting with its predominant macrophage-centric role in solid tumors (8). This immune-context dependency highlights that ID1's mechanism of immunosuppression varies with the prevailing immune cell composition of the TME. Additionally, the signaling pathways upstream of ID1 differ across contexts: in NSCLC, NADK promotes lymph node metastasis by activating the BMP/ID1 axis (22), while in SCLC, NSE drives stemness through the BMP2/Smad/ID1 pathway (47). In hepatocellular carcinoma, BMP9-ID1 signaling promotes EpCAM^+^ cancer stem cell properties, further supporting the conserved role of the BMP-ID1 axis in stemness regulation across malignancies (106). At the post-translational level, hypoxia stabilizes ID1 protein by suppressing the E3 ubiquitin ligase TRIM21, thereby enhancing tumor cell proliferation, migration, and invasion in pancreatic adenocarcinoma (30). Conversely, the deubiquitinase USP1 stabilizes ID1, and its inhibition leads to ID1 degradation and subsequent apoptosis in B-ALL cells, suggesting that targeting ID1 stability is a viable therapeutic strategy (107). MicroRNAs also contribute to ID1 regulation: miR-29b directly targets the ID1 3'-UTR and is repressed by c-Myc downstream of Src signaling, establishing a Src-c-Myc-miR-29b-ID1 axis that controls invasion in lung adenocarcinoma (99). These distinct regulatory inputs further underscore that therapeutic strategies targeting the ID1 axis must be tailored to the specific molecular and cellular context.

The strong rationale for targeting ID1 has led to exploration of diverse strategies, primarily focusing on indirect modulation due to ID1's nature as a transcription factor. These include inhibition of upstream pathways—for instance, using BMP receptor inhibitors such as K02288 or LDN−212854 (106), or natural compounds like sulfated galactoglucan derivatives that bind BMP receptors to block the BMP/Smad/ID1 axis (66); disruption of oncogenic protein complexes, as exemplified by benzaldehyde derivatives that target the 14−3−3ζ/phosphorylated histone H3 interaction to suppress ID1-related transcriptional programs linked to EMT and treatment resistance (108); modulation of protein stability, through USP1 inhibition (107) or the action of Src kinase inhibitors like dasatinib, which can reduce ID1 protein levels (99); and direct suppression via RNA interference (40, 44). Combining ID1 pathway inhibition with immunotherapy is particularly promising given ID1's role in fostering an immunosuppressive TME, such as its expression in TAMs limiting CD8^+^ T cell infiltration (58). Preclinical evidence supports that downregulating ID1 can synergize with immune checkpoint blockade (23). Furthermore, in hepatocellular carcinoma, the ID1/Myc axis promotes oxaliplatin resistance through PD-L1 upregulation and recruitment of immunosuppressive myeloid cells, suggesting that targeting this axis could enhance both chemotherapy and immunotherapy efficacy (59).

However, the development of ID1-targeted therapies faces significant practical challenges. Directly targeting ID1 is pharmacologically challenging because it is a non-enzymatic transcription factor, and potential safety concerns exist due to its roles in normal tissue homeostasis and immune regulation. Moreover, given the compartment-specificity of ID1 functions across tumor and stromal cells (as detailed above), effective therapy may necessitate cell-selective targeting strategies. Therefore, early-phase clinical trials must prioritize safety profiling, pharmacodynamic assessment, and rigorous biomarker-driven patient selection to optimize therapeutic windows and mitigate potential adverse effects.

To provide a concise overview of the clinical landscape surrounding ID1, we have summarized key findings regarding its role as a biomarker, therapeutic strategies under investigation, and remaining challenges in **Supplementary Table 1**.

### 6.3 Future perspectives: from mechanistic insights to context-informed clinical translation

The unequivocal role of ID1 in driving lung cancer aggression and therapy resistance solidifies its status as a compelling therapeutic target. However, its profound context-dependent functionality—acting as either a liability or an asset depending on the cellular compartment (tumor, stromal, or immune) and therapeutic context—demands a paradigm shift from one-dimensional targeting to the development of sophisticated, context-informed precision strategies. To realize this potential and overcome the challenges outlined above, future research must focus on three strategic priorities: (i) defining the predictive contexts for ID1; (ii) developing next-generation functional biomarkers; and (iii) engineering context-informed therapeutics.

A deeper, systems-level understanding of ID1's regulation and function is the cornerstone of future progress. Research must employ integrative multi-omics and single-cell analyses to map the dynamic crosstalk between ID1 and other critical pathways (e.g., TGF-β, VEGF, NF-κB, hypoxia-response) within the specific context of the lung TME. This includes clarifying how upstream signals like hypoxia stabilize ID1 via TRIM21 suppression (30), or how TAM-derived factors (e.g., oncostatin M, TWEAK) activate ID1 through axes such as YAP-ID1 or NF-κB to promote chemoresistance and stemness (53, 73). Importantly, this mechanistic exploration should extend beyond oncology. For instance, in pulmonary arterial hypertension, autophagy activation leads to the downregulation of ID1, thereby promoting vascular cell proliferation (109). This contrast to its stabilization in hypoxic cancer cells highlights the critical, context-dependent regulation of ID1, suggesting that its therapeutic relevance may encompass a broader spectrum of diseases characterized by aberrant cell growth. Critically, this exploration must account for ID1's context-dependent role, as exemplified by the paradoxical finding that upregulating ID1 via promoter demethylation can sensitize hepatocellular carcinoma to sorafenib therapy (110). This underscores the necessity of moving beyond simple ID1 "inhibition" towards strategies that precisely modulate its activity based on the specific tumor biological context. Future studies should also dissect how ID1's compartment-specific functions—distinct in cancer cells versus TAMs, endothelial cells, and other stromal components—can be therapeutically leveraged or restored to re-establish anti-tumor immunity and drug sensitivity.

Translating mechanistic insights into the clinic hinges on the second and third priorities: smarter biomarkers and smarter drugs. Current reliance on ID1 expression levels is insufficient. Future diagnostics must evolve to assess ID1's functional activity—such as its engagement with specific bHLH partner proteins or its role in active protein complexes—within patient samples to reliably predict therapeutic response. Concurrently, the field must engineer context-informed therapeutics. This vision includes: (i) Expanding the arsenal of targeted agents, drawing inspiration from successful interventions in other cancers. Examples include direct ID1 inhibition by natural compounds like Scutellaria flavonoids in NSCLC (111), suppression of ID1 by cannabidiol in diffuse midline glioma (112), disruption of the downstream ID1-Kif11 axis in triple-negative breast cancer (113), and targeting of ID1 within TAMs to remodel the immune microenvironment in colorectal cancer (58); (ii) Designing intelligent delivery systems, such as logic-gated therapies or nanoparticles, that can selectively deliver ID1 modulators to tumor cell subsets or stromal compartments where ID1's role is definitively pro-tumorigenic, thereby overcoming the challenges of compartment-specificity and therapeutic paradox.

The ultimate test lies in rigorous clinical translation. Preclinical evidence, such as trametinib-mediated ID1 downregulation sensitizing KRAS-mutant LUAD to immunotherapy (23), provides a strong rationale for clinical trials testing ID1 pathway inhibitors (e.g., targeting upstream regulators like TRIM21 or the TWEAK/Fn14 axis). Given ID1's central role in angiogenesis, inflammation, and immune suppression, such inhibitors hold particular promise for integration into combinatorial regimens for aggressive disease manifestations. This approach is especially pertinent for MPE, a debilitating complication where current strategies aim to combine local control (e.g., hyperthermic intrathoracic chemotherapy) (114) with systemic agents targeting key pathways like VEGF (14). Given ID1's established involvement in angiogenesis, inflammation, and immune suppression—all processes central to MPE pathogenesis, including the activation of pleural fibroblasts and the fenestration of the pleural vasculature—it represents a hypothesized prime candidate driver and a rational therapeutic target for this condition. Investigating ID1's role in MPE and testing ID1-targeted strategies could disrupt the vicious cycle of vascular leakage and immune suppression in the pleural space. Early-phase clinical trials for any ID1-targeted approach must prioritize safety profiling, pharmacodynamic validation, and rigorous biomarker-driven patient stratification to optimize therapeutic windows.

In conclusion, ID1 stands at a critical juncture in translational oncology. Embracing its complexity through integrated basic and clinical research—focused on defining predictive contexts, developing advanced biomarkers, and engineering precision therapies—is essential to unlock its full clinical potential. This approach will not only advance the fight against lung cancer but may also illuminate therapeutic pathways for associated complications like MPE, moving closer to the promise of truly personalized medicine.

## 7 Conclusion

The accumulating evidence underscores the multifaceted and pivotal role of ID1 in the pathogenesis and progression of lung cancer. From a molecular perspective, ID1 emerges as a key regulator that drives tumor cell proliferation, invasion, metastasis, and stemness maintenance. Its profound influence extends beyond cancer cells, actively remodeling the TME by modulating immune cell dynamics, angiogenesis, and stromal interactions. This integrative function positions ID1 as a critical liaison between intrinsic tumor aggressiveness and extrinsic microenvironmental support, contributing significantly to therapeutic resistance and poor clinical outcomes.

A compelling and translational aspect explored in this review is the potential involvement of ID1 in MPE formation. While direct experimental and clinical evidence definitively linking ID1 to MPE pathogenesis remains limited, the strong mechanistic overlap between ID1's established functions—orchestrating angiogenesis, inflammation, immune evasion, and stromal crosstalk—and the core pathophysiology of MPE presents a rigorously testable hypothesis. ID1 may act as a key molecular node linking primary lung tumor biology to the development of this debilitating complication, offering a novel conceptual framework for future investigation; however, this possibility awaits direct experimental confirmation, and we emphasize that this connection should be regarded as a hypothesis rather than an established mechanism.

The clinical translation of ID1 biology is marked by its context-dependent duality, functioning as both a driver of resistance and a potential sensitizer to specific therapies. This complexity underscores that ID1 is more than a simple prognostic biomarker; it is a dynamic component of tumor adaptation. Targeting the ID1 axis, therefore, requires sophisticated, context-informed strategies rather than unilateral inhibition.

Future research efforts should be prioritized in several key directions. First, mechanistic dissection through single-cell and spatial omics technologies is needed to delineate cell-specific ID1 functions within the lung TME and the pleural space. Second, therapeutic innovation must focus on developing agents that target ID1 or its key regulators and testing their efficacy, particularly in combination with immunotherapy or anti-angiogenic agents, in advanced disease and MPE settings. Third, biomarker refinement should move beyond static expression measurements toward functional assays that capture ID1 activity, enabling better prediction of patient subsets most likely to benefit from ID1-directed therapies.

In summary, ID1 stands at the convergence of multiple oncogenic pathways, serving as a key regulator of lung cancer malignancy and a promising, albeit complex, therapeutic target. Unlocking its full clinical potential—particularly for challenging sequelae such as MPE, a link proposed in this review—will necessitate a multidisciplinary approach that bridges fundamental biology, translational tool development, and precision clinical trial design. Ultimately, the goal of such endeavors is to translate these intricate molecular insights into tangible clinical benefits, improving survival and quality of life for lung cancer patients.
