# Supplementary material for: Inhibitor of DNA binding 1 in lung cancer and the tumor microenvironment: a testable hypothesis for malignant pleural effusion formation
Source: Front Immunol. 2026 Jul 22;17:1743032. doi: 10.3389/fimmu.2026.1743032 (PMC13437535; doi:10.3389/fimmu.2026.1743032)
Supplement: Supplementary file 1 [file Table1.docx]

****Supplementary Table 1. Clinical significance of ID1: biomarkers, therapeutic strategies, and challenges****

| **Category** | **Key Findings** | **References** |
| --- | --- | --- |
| ****Prognostic biomarker**** | • High ID1 expression is an independent prognostic factor for shorter survival in NSCLC patients with adenocarcinoma histology | 97 |
|  | • NADK activates ID1 expression via the BMP pathway to promote lymph node metastasis in NSCLC, associated with poor prognosis | 22 |
|  | • Co-expression of Id1 and Id3 correlates with poor clinical outcome in stage III-N2 NSCLC patients treated with definitive chemoradiotherapy | 39 |
|  | • The NSE/BMP2/Smad/ID1 axis drives stem cell-like characteristics in SCLC, correlating with poor patient prognosis | 47 |
|  | • miR-381 targets and represses ID1; its downregulation in lung adenocarcinoma correlates with poor prognosis | 98 |
|  | • miR-29b is involved in the Src-ID1 signaling pathway and is downregulated in lung adenocarcinoma, serving as a potential prognostic marker | 99 |
| ****Predictive biomarker**** | • ID1 mediates resistance to osimertinib in EGFR T790M-positive NSCLC through EMT | 40 |
|  | • ID1 overexpression increases sensitivity to gefitinib via activation of necroptosis | 101 |
|  | • ID1 is associated with trametinib resistance; its downregulation enhances sensitivity to PD-1/PD-L1 blockade in KRAS-mutant LUAD | 23 |
|  | • ID1 expression inversely correlates with PD-L1; ID1 blockade enhances the antitumor efficacy of PD-1 inhibitors | 57 |
| ****Therapeutic strategies**** | ****Indirect targeting (upstream pathways)**** |  |
|  | • BMP receptor inhibitors (e.g., K02288) block BMP/Smad/ID1 signaling | 66, 106 |
|  | • MEK inhibitor trametinib downregulates ID1 via the proteasome-ubiquitin system | 23 |
|  | • USP1 inhibitor induces apoptosis in B-ALL cells via the ID1/AKT pathway | 107 |
|  | • Targeting 14-3-3ζ overcomes EGFR-TKI resistance through BMP2/Smad/ID1 signaling | 100 |
|  | • Targeting the TRIM21-ID1 axis counteracts hypoxia-induced pancreatic cancer progression | 30 |
|  | • TWEAK/Fn14 signaling promotes ID1 expression via NF-κB, implicating intervention in hepatocarcinogenesis | 53 |
|  | • TAM-secreted OSM activates the YAP-ID1 axis, driving cholangiocarcinoma progression and chemoresistance | 73 |
|  | • Chemotherapy upregulates ID1 in endothelial cells of SCLC, enhancing angiogenic activity | 65 |
|  | • 5-aza upregulates ID1 by inhibiting promoter methylation, enhancing sorafenib efficacy in hepatocellular carcinoma | 110 |
|  | • Targeting the ID1/STAT3/ATF6 autophagy pathway overcomes chemoresistance in ovarian cancer | 103 |
|  | ****Direct targeting of ID1**** |  |
|  | • RNA interference directly silences ID1 | 40, 44 |
|  | • Scutellaria flavonoids inhibit ID1 to suppress malignant phenotypes in NSCLC | 111 |
|  | • Cannabidiol inhibits ID1, reducing invasion and tumor growth in diffuse midline glioma | 112 |
|  | • Benzaldehyde derivatives target the 14-3-3ζ/H3S28ph interaction, suppressing ID1-related transcriptional programs | 108 |
|  | • Small-molecule inhibitor targeting the ID1-Kif11 axis (ispinesib) combined with chemotherapy for triple-negative breast cancer | 113 |
|  | • Targeting ID1 in TAMs reshapes the immune microenvironment in colorectal cancer | 58 |
| ****Combination strategies**** | • Trametinib + PD-1/PD-L1 blockade shows synergistic effects in KRAS-mutant LUAD | 23 |
|  | • Id1 blockade + PD-1 blockade enhances antitumor immunity | 57 |
|  | • Targeting the ID1/Myc axis overcomes oxaliplatin resistance and enhances immune response in hepatocellular carcinoma | 59 |
|  | • Ispinesib + chemotherapy for triple-negative breast cancer | 113 |
| ****Challenges**** | • **Functional duality:** ID1 can mediate resistance (osimertinib) or enhance sensitivity (gefitinib, sorafenib) depending on drug and cellular context | 40, 101, 102 |
|  | • **Cell-type specificity:** ID1 exerts distinct functions in tumor cells, TAMs, and endothelial cells | 73, 65, 58 |
|  | • **TME heterogeneity:** ID1 function is influenced by immune cell composition, hypoxia, and other TME factors | 8, 30, 58 |
|  | • **Molecular subtype differences:** ID1 plays different roles in SCLC; its role in lung squamous cell carcinoma remains unclear | 47 |
| ****Clinical trials / translational studies**** | • HITHOC combined with TKIs for lung cancer with MPE | 114 |
|  | • VEGF-targeted therapy (bevacizumab, apatinib) combined with cisplatin for NSCLC with MPE (preclinical study) | 14 |

Abbreviations: B-ALL, B-cell acute lymphoblastic leukemia; EGFR, epidermal growth factor receptor; EMT, epithelial-mesenchymal transition; HITHOC, hyperthermic intrathoracic chemotherapy; ID1, inhibitor of differentiation/DNA binding 1; LUAD, lung adenocarcinoma; MPE, malignant pleural effusion; NADK, nicotinamide adenine dinucleotide kinase; NSCLC, non-small cell lung cancer; PD-1, programmed cell death protein 1; PD-L1, programmed death-ligand 1; SCLC, small cell lung cancer; TAM, tumor-associated macrophage; TKI, tyrosine kinase inhibitor; TME, tumor microenvironment.
